# The gut microbiome molecular mimicry piece in the multiple sclerosis puzzle

**DOI:** 10.3389/fimmu.2022.972160

**Published:** 2022-08-15

**Authors:** Noha S. Elsayed, Paula Aston, Vishnu R. Bayanagari, Sanjay K. Shukla

**Affiliations:** ^1^ Center for Precision Medicine Research, Marshfield Clinic Research Institute, Marshfield, WI, United States; ^2^ Department of Neurology, Marshfield Clinic Health System, Marshfield, WI, United States

**Keywords:** multiple sclerosis, gut microbiome, molecular mimicry, microbial metabolites, aberrant immune response, leaky gut, HLA genes, Epstein-Barr virus

## Abstract

The etiological complexity of multiple sclerosis, an immune-mediated, neurodegenerative disease with multifactorial etiology is still elusive because of an incomplete understanding of the complex synergy between contributing factors such as genetic susceptibility and aberrant immune response. Recently, the disease phenotypes have also been shown to be associated with dysbiosis of the gut microbiome, a dynamic reservoir of billions of microbes, their proteins and metabolites capable of mimicring the autoantigens. Microbial factors could potentially trigger the neuroinflammation and symptoms of MS. In this perspective article, we discussed how microbial molecules resulting from a leaky gut might mimic a host’s autoantigen, potentially contributing to the disease disequilibrium. It further highlights the importance of targeting the gut microbiome for alternate therapeutic options for the treatment of MS.

## Introduction

Multiple sclerosis (MS) is an immune-mediated, chronic, debilitating, demyelinating neurodegenerative disease. It is estimated to be affecting 2.8 million people worldwide in 2020, a 30% increase since 2013, and in the United States, MS patients number roughly 400,000 people (1–3). MS is a complex autoimmune disease in which the combined roles of genetic susceptibility and aberrant immune response are largely established. However, an understanding of how environmental or microbial factors, infectious or not, initiate, trigger, or maintain the different stages or phenotypes of MS remains to be established. Despite the availability of several therapeutic treatments over the last few years, mainly to stall the disease’s progression, still there is no cure for MS. Failure to develop a cure stems partially from an incomplete understanding of MS pathophysiology and the factors that trigger its symptoms. This article attempts to piece together these pieces of the MS jigsaw puzzle.

The pathogenesis of MS is considered secondary to the autoreactive lymphocytes entering the central nervous system and causing neuroinflammation and subsequent demyelination of the axons (4, 5). The hallmark of this disease is axon demyelination where muscle weakness, blurred vision, and malfunctioned urinary and gastrointestinal systems are its visible consequences (6). Clinically, the disease has four phenotypes: clinically isolated syndrome (CIS), relapsing remitting MS (RRMS), secondary progressive MS (SPMS), and primary-progressive MS (PPMS), the latter of which can be further classified into active and inactive/remission (7). A majority of patients (85%) suffer from RRMS, but others can convert to a progressive course with an accumulation of disabilities leading to the loss of mobility (8). PPMS is a steady progressive form of MS from its onset (9). The heterogeneity of the MS symptoms, its undefined pathogenesis, unknown triggering factors, and its different phenotypes pose a challenge for the diagnosis and treatment of this debilitating disease.

## Genetics of MS

There is a strong genetic component to MS as evidenced by its higher concordance rate in monozygotic twins (25-30%). However, it has a lower rate in dizygotic twins (3-7%) and first-degree relatives (3%). Several familial studies supported the presence of a genetic component in MS, but the rapid decrease in risk from monozygotic twins to other family members illustrates a polygenic nature (10–12). The polygenic risk of MS is both concentrated and dispersed consistent with its spectrum of symptoms. Indeed, no single genetic variant can predict effectively the susceptibility of patients (13). Genetic risk (11) increases with the increasing number of risk alleles in a patient (14). Many studies have investigated the genetic architecture of MS disease; however, the results were not always replicated. This is consistent with the common disease common variant theory which implies that common diseases are caused by hundreds of common variants, each of which has a small effect (11, 15). The most definitive genetic association to MS disease is the human leukocyte antigen genes (HLA) class II or more narrowly DRB1* 15:01 allele (11, 16, 17) on chromosome 6p21 (18) which links the adaptive immune system to MS disease pathogenesis. However, the DRB*15:01 allele has a moderate effect in predicting disease susceptibility (19) and seems to be associated with both the disease’s early onset in general and with a high disease rate in women (17, 18). Other studies reported an association between HLA-DRB1*04 and PPMS clinical phenotype (20, 21). However, this association with PPMS has not been confirmed by other studies (18, 22, 23). Other HLA alleles such as HLA-A*02:01 and HLA-DRB1*11 showed a protective role for MS (24, 25). Roughly, 200 genetic loci residing in the non-HLA polymorphic genes have also been implicated, accounting for 20% of the MS genetic associations. The majority of these non-HLA alleles are related to immunogenic pathways such as interleukin 7 receptor (IL7R) (26), interleukin 2 receptor (IL2RA) (27), *TAGAP* (28), and vitamin D receptor (VDR) (29). These variants associated with non-HLA genes affect the function of their respective genes, such as how the creation of soluble protein of both IL2 and IL7 genes leads to downstream signaling inhibition (11, 30).

## Immunology of MS

MS is a disease of aberrant immune signaling pathways modulated by hundreds of genetic variants regulating immune cells. In terms of immuno-pathophysiology, MS is linked to the imbalance between T regulatory (Treg), and T helper (Th) cells (31). Th cells usually recognize the presented peptides by major histocompatibility complex (MHC) class II on the antigen presenting cells (32). Th cells differentiate into Th1, Th2, and Th17, and these differentiated cells secrete different cytokines such as IL17, TNF-α, IFN-γ, IL-4 and CCL4 (33). Treg cells are the only self-reactive T-cells to maintain immune homeostasis. They normally express interleukin (IL2) receptor α chain (CD25) and its function depends on the transcription factor FOXP3 (34). Treg cells produce inhibitory cytokines such as IL10 and transforming growth factor (TGF)-β. These cytokines inhibit the proliferation of inflammatory Th cells and promote immune tolerance which protects against demyelination (35).

MS patients have abundant inflammatory Th17 cells and low levels of the anti-inflammatory Treg cells. This immune imbalance enhances the infiltration of monocytes and macrophages (35, 36) in central nervous system (CNS) which increases the reactive oxygen species, triggers the lesion formation, disturbs the tight junctions of the blood brain barrier (BBB), and attracts more monocytes towards the BBB. This eventually leads to phagocytosis of the myelin and the loss of the action potential (37, 38). In summary, overactive T cells start the RRMS and the progressive state is maintained by the infiltrating monocytes and macrophages in the CNS (39). Interestingly, Venken et al. (2006) showed that there was no difference in Tregs count between RRMS and secondary progressive, the two phenotypes of MS disease (40). Lately, the role of B cells was studied for MS pathology where the cells were displaying a proinflammatory phenotype in patients, exacerbating the Th17 cell response (41–43). The host genetics comprises one piece of the puzzle, but leaves questions regarding possible links between genetic susceptibility, MS immune-pathophysiology, microbial agents or their products from gut and/or outside environments.

These questions linger despite several studies reporting associations or interactions between the host’s genetic susceptibility and environmental factors, such as smoking (44), obesity (45), heavy metal poisoning (46), low vitamin D, high salt intake (33, 47), and Epstein-Barr virus infection (48) - all have a reported association with MS. Of particular note, it has been suggested that a related low exposure to infectious agents in childhood helps prime the immune system (49). An emerging theme for microbial triggers comes from the rich microbial reservoir of the human gut. Indeed, the importance of human gut microbiome cannot be overstated as any one or more of its thousands of viruses or bacterial species could potentially house one of MS’s triggers. This enormous repertoire of microbial factors and metabolites has been shown to possess a path connecting the gut-brain nexus (50).Furthermore, the gut microbiome dysbiosis has demonstrated associations with other complex autoimmune and neurological diseases such as Alzheimer’s disease, Parkinson’s, and autism spectrum disorder (51–53). If microbial triggers from gut microbiome, especially its microbial peptides, can be a source of molecular mimicry with the myelin sheath, then this could potentially explain, in part, how the MS is triggered in genetically susceptible individuals.

## Gut microbiome and MS

The search for an elusive, consistent infectious agent with a triggering or contributing role in causing MS has led to a much-needed investigation of dysbiosis in human gut microbiota. The plausibility of such an agent lies in the availability of billions of microbes whose proteins or metabolites could interact with a host’s signaling pathways to determine the overall health of a person. Certainly, gut microbe(s) could trigger the neuroinflammation and the symptoms of MS. Indeed, numerous studies have reported dysbiosis of the gut microbiome in MS cohorts capable of either a triggering or a facilitating infectious agent mechanism for developing MS in genetically susceptible individuals compared to non-MS subjects (54–57). Moreover, a relapsing-remitting, mouse MS model (experimental autoimmune encephalomyelitis (EAE)) suggests a triggering of MS disease upon exposure to commensal, but not pathogenic, gut bacterial flora (58). Even individual bacterium from the gut microbiome (specifically, segmented filamentous bacteria) was capable of inducing EAE in germ-free mice (55). Consistent with a role for gut microbiota in MS, treatment of the EAE experimental mice with non-absorbing oral antibiotics, kanamycin, colistin, and vancomycin for reducing the bacterial load in the gut led to the improvement of EAE development in those mice (33). The administration of antimicrobials and resulting changes in the gut microbiota further lead to the reduction of proinflammatory cytokines and mesenteric Th17 cells (59), which has been reported to play a role in MS disease development. Despite this evidence, MS risk cannot be efficiently reduced by antibiotics alone (60); many pieces of the gut microbiome puzzle related to MS disease are still missing.

In MS patients, gut microbiome dysbiosis has been reported at different taxa level (61–63) (**Table 1**). Understanding these perturbations in MS patient’s vis-à-vis non-MS healthy controls from a similar environment can shed light on the function of one or more taxa in MS. For instance, *Methanobrevibacter* has been reported to be in higher relative abundance in the gut microbiome of MS patients as displayed in **Table 1** (56) as well as in patients with other inflammatory autoimmune diseases (73). It has been proposed that *Methanobrevibacter* may play a complex role, activating the dendritic cells and recruiting other inflammatory cells, as well as producing methane gas, which delays the gut transit time leading to constipation, one of the MS symptoms (63, 74). On the other hand, the depletion of *Bacteroides fragilis* and *Clostridioides* in gut microbiota of MS patients as shown in **Table 1** suggests a protective function in healthy individuals (63). For instance, the polysaccharide A of *B. fragilis* protected against axon demyelination in mice (75), and the *Clostridioides* produce butyrate, which increases the Treg differentiation favoring the anti-inflammatory state (54, 76). Then, why not there is a MS gut microbiome biosignature identified from the dysbiosis from MS patients? It is because not all studies have observed major changes between the gut microbiome in MS and healthy control except that *Akkermansia* and *Methanobrevibacter* showed consistent increase in MS patients while *Prevotella* and *Bacteroides* were reduced in MS patients (67). Furthermore, Knight et al. (2014) reported that predicting discrete clusters as disease biomarkers is not highly effective because the human microbiome is a continuous gradient of taxa (77) besides being dynamic, subjected to transient changes due to non-disease associated factors such as diet, exercise and medications. In addition to bacterial dysbiosis, changes in the relative abundance of specific fungi have also been reported (**Table 1**). In summary, detection of precise microbial signature for MS is still elusive and needs to be evaluated longitudinally during the disease course and from multi-ethnic patients living in different environmental conditions.

**Table 1:** Reported changes in gut microbiome of MS patients cohorts.

Organisms	Increase	Decrease	References
Phylum	Firmcutes Proteobacteria Euryarchaeota Verrucomicrobia Basidiomycota*	Bacteroidetes Ascomycota*	(56, 64, 65)
Family	*Lachnospiraceae* *Desulfovibrionaceae*		(63, 66)
Genus	*Methanobrevibacter Akkermansia* *Streptococcus* *Aceintobacter* *Blautia* *Bifidobacterium* *Aldercreutzia* *Flavobacterium* *Pseudomonas* *Mycoplana* Saccharomyces* Aspergillus * Candida* Epicoccum*	*Butyricimonas* *Clostridia* *Preveotella* *Bacteroides* *Lactobacillus* *Sutterella* *Collinsella* *Coprobacillus* *Anaerostipes*	(54, 56, 57, 64, 65, 67–71)
Species	*Akkermansia muciniphila*	*Preveotella histicola* *Bacteroids fragilis* *Parabacteroides distasonis*	(57, 62, 63, 72)

*Fungi.

## Microbiome metabolites and MS

Could microbiome-produced metabolites be identified as biomarkers in MS? Lower concentration of lipid 654 produced by *Bacteroidetes* species is being explored as a biomarker as this compound showed decrease in concentration in both MS and Alzheimer patients in comparison to healthy controls (78). Jangi et al. (2016) reported an in increase in breath methane in MS patients, which may be corresponding to the increase of *Methanobrevibacter* in gut microbiome (56). During dysbiosis, the microbiota either sequester important nutrients from the host or alter the production of metabolites. The former mechanism is shown with the taxa *Desulfovibrionaceae*, which sequester cysteine. This amino acid is mostly used for the synthesis of glutathione, a tripeptide of cysteine and glutamate, protect against reactive oxygen species in the CNS (37). As a consequence, a low concentration of glutathione has been encountered in MS patients in comparison to heathy individuals (79). The latter mechanism for dysbiosis is the altered metabolite production in favor of low beneficial compounds or high detrimental ones. Another example of beneficial metabolites are the short chain fatty acids (SCFAs) like acetate, butyrate, and propionate. Collectively, the amount and type of SCFAs produced depend on the microbiota composition and the substrates available (80). SCFAs are produced by a wide variety of gut bacterial species such as *Bacteroides, Bifidobacterium, Lactobacillus, and Clostridium* (35). Bacteroidete*s* produce acetate and propionate, while firmicutes produce butyrate (33). Many studies pointed out the beneficial effects of SCFA for MS. SCFAs maintain the integrity of both the intestinal barrier through increasing the expression of tight junction proteins (35, 76) and the BBB (81). Moreover, SCFAs increase the differentiation of the anti-inflammatory Treg, which can relieve the axonal damage to some extent (35). Additionally, SCFAs could inhibit histone deacetylases in a concentration-dependent manner as it helps maintain immune homeostasis (82). Furthermore, propionate has been suggested as a potent immunomodulatory for MS patients which decreases the relapse episodes and brain atrophy because it increases Treg and decreases Th17 cells (83). In MS patients, both stool and plasma revealed a decrease in SCFAs concentration which implies a reduction in SCFA-producing bacteria of the gut microbiome (7, 54, 68). Additionally, the higher level of *Prevotella* in MS patients undergoing disease-modifying therapy was accompanied by higher concentration of butyrate (84). In conclusion, SCFAs are crucial for establishing an anti-inflammatory state, a state usually deficient in MS patients.

Gut microbiota such as firmicutes, *Lactobacillus*, and *Enterobacteriaceae* (to name a few) can metabolize aromatic amino acids such as tyrosine, phenylalanine, and tryptophan. Mostly the genera in phylum firmicutes can produce P-cresol from tyrosine and phenolic compounds from phenylalanine (35). Through the kynurenine pathway, they can also metabolize tryptophan into different metabolites known as “TRYP-6.” These metabolites can be kynurenine, quinolinate, indole, indole acetic acid (IAA), indole propionic acid (IPA) and tryptamine (35). Some of these compounds, namely indole derivatives, can cross the BBB and act on aryl hydrogen receptor (AHR), further decreasing reactive oxygen species and controlling the neuroinflammation processes (85). Moreover, other tryptophan metabolites, after binding to AHR receptors, exhibit anti-inflammatory effect on astrocytes. The astrocytes are the main component of neurovascular units, which preserve the integrity of BBB under normal conditions (86, 87). However in MS, it was observed that astrocytes upregulates 4-galactosyltransferase enzymes, which boost inflammation of the CNS (88). Thus, the anti-inflammatory effect of tryptophan metabolites helps delay the progression of MS disease (89). Other studies reported variations in the kynurenine level in different stages of MS disease (active/inactive) and clinical phenotypes (90). Considering all these complex interactions, the challenge remains to fully understand the exact mechanism of tryptophan metabolism in MS. Other metabolites such as *Bacillus*-derived poly-gamma-glutamic acid favor the imbalance of Th cells towards Th1 rather than Th17 cells (91). Phytoestrogens metabolites have anti-inflammatory effect and a significant amelioration of EAE in mouse model (92). Bacteria metabolizing phytoestrogens such as *Prevotella* and *Aldercreutzia* are decreased in MS patients, which would favor the inflammatory state in the MS patients. Thus, the metabolites produced by gut microbiome could affect the intestinal barrier integrity, the immune tolerance, and the neuroinflammation encountered in the brain.

## Could microbial molecular mimicry trigger MS?

Microbes-derived molecular mimicry emanating from the homology between microbial- and human-derived antigens has been known to induce autoimmunity (93–95). In rheumatic fever, molecular mimicry is displayed between M proteins of *Streptococcus pyogenes* and the cardiac myosin (96). Mechanistically, a cross reactivity could happen when T cells recognize antigens through major histocompatibility complex (MHC) class II *via* a sequence of 8-10 amino acids (97),- a small enough number of amino acids that can be shared between microbial and host peptides-and/or due to the similarity of anchor proteins that bind specifically to MHC molecules, a phenomenon known as polyspecificity. The MHC usually binds to these anchor amino acids residues but has flexibility in the remaining residues, a flexibility that increases the responsiveness of MHC class to variety of pathogens and xenobiotics (98). In the case of MS, amino acid changes encountered in the MHC binding peptides affect those antigens binding to the HLA-DRB1 (11). Thus, the molecular mimicry of bacterial peptides to the myelin protein are worth experimental exploration. Hundreds of thousands of proteins from thousands of bacterial species and their serovars from gut microbiota could potentially produce autoantigenic peptides in genetically susceptible individuals or under certain immunodeficient conditions. For instance, different bacterial species of the gut microbiome can induce imbalance in Th and Treg cells (55, 58). Moreover, for T cells to attack the myelin sheath inside the CNS, they should be activated peripherally by maybe a bacterial peptide (99). In the brain of MS patients, it has been reported that bacterial peptidoglycan in antigen-presenting cells suggests a triggering of the pathophysiology through bacterial products (100). Similarity between bacterial products and autoantigens capable of inducing MS has also been studied. Mostly, the primary candidates autoantigens were myelin basic protein (MBP), proteolipid protein, myelin-associated glycoprotein, and myelin oligodendrocyte glycoprotein (101). However, MBP was thoroughly investigated because of its induction of MS symptoms in mice and genetically susceptible primates in presence of an adjuvant (102, 103). The MBP sequence was divided into the tryptophan, midpeptide, and hyperacute regions (103, 104). Some proteins of *Bacteroides* and *Bifidobacterium* species showed some similarity with different MBP regions (103). Interestingly, even the adjuvant required for induction of the immune response is found in bacteria like *N*-acetylmuramyl dipeptide (103). Still the question remains: why does MS happen in certain patients when these antigens and adjuvants are available in every human gut?

Furthermore, CD4 T cells can be activated by another candidate, GDP-L-fucose synthase, in a manner quite similar to the myelin sheath especially in HLA-DRB3* positive patients. Fucose synthase peptides have homology with bacterial fucose synthase of *Akkermensia*, and *Prevotella* (99). Not only with specific bacterium, cross reactivity between the myelin sheath and the Epstein-Barr virus nuclear antigen 1 has also been shown suggesting Epstein-Barr virus as a possible inducer of MS especially in genetically susceptible people (48, 105, 106). Surprisingly, when *Acinetobacter* and *Pseudomonas* act as infectious agents they use mucono-decarboxylase enzyme, which shares a similar sequence with myelin protein (107). These in-silico and experimental studies present a plausible relationship between MS autoimmune targets and molecular mimicry emanating from microbes, possibly gut microbes. The molecular mimicry emanating from the gut microbiome has also been investigated in another CNS demyelinating disease, neuromyelitis optica (108), an autoimmune disease causing optic neuritis and active myelitis. In neuromyelitis optica, autoantibodies are produced against the astrocyte water channel protein aquaporin 4 (AQP4) (109). Interestingly the commensal bacterium, *Clostridium perfringens* showed increased relative abundance in the patients of neuromyelitis optica (110) than the healthy controls and the adenosine triphosphate-binding cassette (ABC) transporter permease sequences of C. *perfringens* shared homology with T cell epitope with the Aquaporin 4 with the possibility of mimicry from the gut (108). However, it should be noted that induction of autoimmune disease by molecular mimicry is complex and bacterial or viral peptide(s) acting alone are expected to be insufficient to initiate a complex disease like MS. There ought to be accompanying host genetic susceptibility linked with aberrant immune response triggered by one or more microbial antigens or molecules. And just like the dispersed genetic variants associated host genetic susceptibility, peptides from more than one microbe could be capable of mimicry in different MS patients.

## The leaky gut and translocation of gut microbial peptides

The MS-gut microbiome association operates with an underlying assumption that one or more microbial triggers from the gut can cross the intestinal barrier due to a phenomenon called leaky gut which allows dissemination of the bacteria to distal organs and enables immune system exposure to such bacteria (76). Disruption of the intestinal barrier could be due to dysbiosis; indeed, *Akkermansia* species could feed on the mucin layer in the mucous layer of the intestine, exposing the gut microbiome to systemic circulation (56, 111). Alternatively, MS patients exhibit high secretion of INF-γ, which would stimulate the differentiation of Th cells to Th17 cells. Th17 cells produce IL17, which in combination with INF-γ would reorganize the intestinal tight junctions leading to a compromised barrier (112). Again, this barrier disruption would expose the already hidden gut microbiome to the immune system. A disturbed intestinal barrier is usually evaluated by the lactulose/mannitol permeability test, and in case of MS patients, abnormal permeability has been reported in about 73% of MS patients (113). In animal models, the severity of EAE increased with compromised intestinal barrier but improved with treatment with *Escherichia coli* strain Nissle 1917 as a probiotic (114) suggesting ameliorating intestinal barrier could be one of the potential goals for innovative treatment to attenuate the MS disease.

## Immunologic tolerance

The gut microbiota are important for achieving immunologic tolerance and establishing good immune response against pathogens. This was partly confirmed by the upregulation of CD+4 T cells and development of gut-associated lymphoid tissues in the underdeveloped immune system of germ-free mice after the transfer of commensal bacteria (115). The gut immune cells do not respond to commensal bacteria because of a special phenotype of macrophage called “inflammation anergy” (116). Moreover, any inflammatory response in the gut is usually suppressed by cytokines produced by lymphocytes in the gut-associated lymphoid tissues (86). As mentioned before, the imbalance between Th17 and Treg is one of the studied causes for MS disease. Interestingly, Th17 and Treg cells are found in the intestine where gut microbiome is assumed to enhance the differentiation of Th17 (117). The germ-free mice do not have Th17 except after induction of microbial colonization (118). On the other hand, no significant difference in Treg counts between MS patients and healthy controls was observed (40, 119). However, the Tregs showed lower antinflammatory function in MS patients through reduction in IL10 production in germ free mice transplanted with fecal samples of MS patients in comparison to that of healthy control (57). The colonic Treg cells also depend on some microbial signals for efficient function (120). For instance, CD41FOXP31 Treg cells are upregulated by *Clostridia* (*Clostridioides*) strains (121) and by *P. histicola* in mice which led to downregulation of IL-17 and IFNγ (62). Moreover, the gut microbiota do not affect only other immune cells numbers but their function. For example, the capacity to kill pathogens of neutrophils was reduced in germ free mice (122).

## Discussion

**Figure 1:**
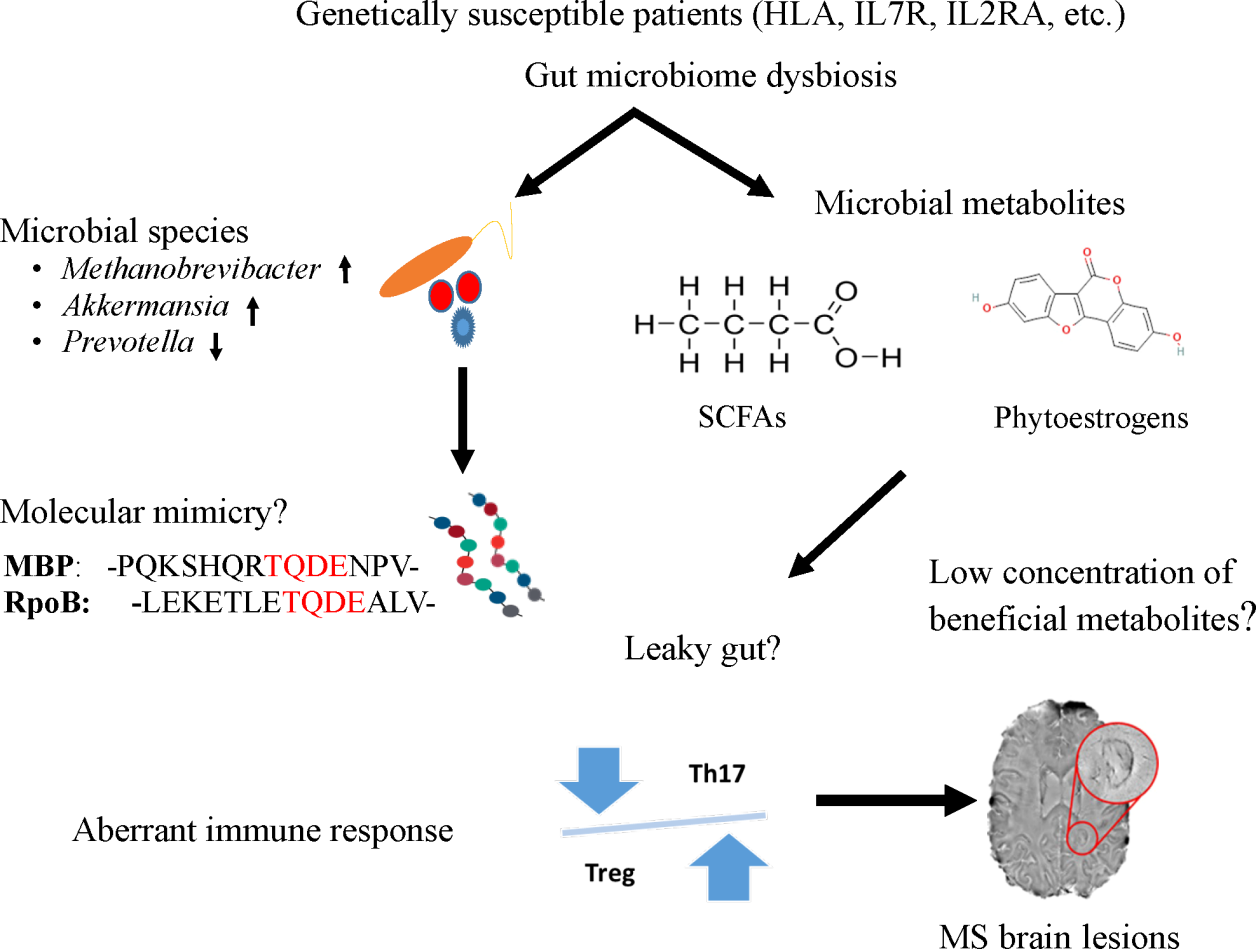
A potential mechanism to link the role of gut microbiome to trigger and maintain MS symptoms in genetically susceptible patients. Specific microbes (e.g., Methanobrevibacter and Akkermansia), microbial peptides [RNA polymerase B (103)], or metabolites (short chain fatty acids and phytoestrogens) from the gut could enter the circulatory system due to leaky gut and induce aberrant immune response, particularly the imbalance of Th and Treg cells. The T cells imbalance where Th17 cells outnumber Tregs impairing immune tolerance and subsequently lead to inflammation and MS lesions in the brain.

As a heterogeneous, complicated, immune-mediated disease, MS has complex etiology. Several studies have provided evidence that the gut microbiome plays a role in MS pathogenesis, therefore it could help in its diagnosis, and targeted therapeutic intervention with further research. In this article, we highlighted the current understanding of the potential roles of microbial agents or their products from the gut microbiota in MS as displayed in **Figure 1**. We speculated that either molecular mimicry or microbial metabolites in the gut microbiome could contribute to the known aberrant immune response of Th17 and Treg imbalances. Many studies have reported compositional alteration of different microbial species of gut microbiome in MS. Indeed, the gut microbiome with its rich microbial reservoir and their protein could be a source for faulty cross-recognition of Th cells, leading to this aberrant immune response. Moreover, the plentiful anti-inflammatory metabolites produced by the gut microbiome suffer from a reduction in MS diseases potentiating the inflammatory state encountered in this disease. With the puzzle thus far presented, there is a need to further understand these complex relationships between, the genetics, immune response and the gut microbiome to understand the pathophysiology of the disease and develop better therapeutic options for this debilitating disease.

## Author contributions

NSE: conceptualization, writing the original draft article, and final editing. PA: review and editing, VRB: conceptualization and review, SKS: conceptualization, writing, reviewing, and final editing. All authors contributed to the article and approved the submitted version.

## Funding

This study was funded, in part, by Physician-Scientist Collaboration Research award from MCRI to SKS and PA by Project Number 441190-00. NE was supported by Ebenreiter Post Doctoral fellowship award (500430-00 ) in precision medicine. 

## Acknowledgments

We would like to thank Dr. David Puthoff for his editorial assistance and Dr. Joe Mazza for reviewing the manuscript.

## Conflict of interest

The authors declare that the research was conducted in the absence of any commercial or financial relationships that could be construed as a potential conflict of interest.

## Publisher’s note

All claims expressed in this article are solely those of the authors and do not necessarily represent those of their affiliated organizations, or those of the publisher, the editors and the reviewers. Any product that may be evaluated in this article, or claim that may be made by its manufacturer, is not guaranteed or endorsed by the publisher.
